# Evaluation of Time and Temperature Storage Effects on Canine Blood Anticoagulated with Citrate-Phosphate-Dextrose-Adenine Supplemented with Ethylenediaminetetraacetic Acid: Investigation of Optimal Conditions for External Quality Assessment Samples

**DOI:** 10.3390/ani16142170

**Published:** 2026-07-13

**Authors:** Sakurako Neo, Shoichi Sato, Yukihiro Fukuda, Sara Kurokawa, Kana Kobayashi, Masaharu Hisasue, Reina Segawa, Kensuke Saito

**Affiliations:** 1 Laboratory of Clinical Diagnosis, School of Veterinary Medicine, Azabu University, Sagamihara 252-5201, Japan; 2 Clinical Engineering, Faculty of Medical Science, Juntendo University, Urayasu 279-0013, Japan; s.sato.aq@juntendo.ac.jp; 3 Department of Clinical Laboratory, Funabashi Municipal Medical Center, Funabashi 273-8588, Japan; fukuda_yu@mmc.funabashi.chiba.jp; 4 Physiological Function Testing Department, Eastern Chiba Medical Center, Togane 283-8686, Japan; kurokawa.s.515@gmail.com; 5 Department of Laboratory Medicine, National Hospital Organization Sendai Medical Center, Sendai 983-8520, Japan; kobayashi.kana.df@mail.hosp.go.jp; 6 Laboratory of Small Animal Internal Medicine, School of Veterinary Medicine, Azabu University, Sagamihara 252-5201, Japan; hisasue@azabu-u.ac.jp; 7 Bio & Healthcare Field Department, Sales Promotion Center, Sales Division, HORIBA Ltd., Kyoto 601-8150, Japan; reina.segawa@horiba.com; 8 Business Planning Department, Bio & Healthcare Technology Division, HORIBA Ltd., Chiyoda 101-0063, Japan; kensuke.saito@horiba.com

**Keywords:** canine blood, CPDA-1, EDTA2-K, external quality assessment storage temperature

## Abstract

External quality assessment (EQA) using fresh blood from companion animals has not yet been conducted worldwide. This study investigated storage temperature, duration, and anticoagulant conditions to identify more stable sample preparation methods for fresh canine blood stored for more than 24 h, as required for distributed surveys. Blood samples from five healthy dogs containing CPDA-1 supplemented with EDTA-2K at final concentrations of 1, 2, and 3 mg/mL were stored at room temperature (RT group) and refrigerated temperature (RE group). Complete blood count measurements were performed at 2, 5, 8, 24, 48, and 72 h after blood collection using the Microsemi LC-662, a point-of-care testing (POCT) automated hematology analyzer. White blood cell and platelet counts were more stable over time in the RT group compared with the RE group. In contrast, red blood cell parameters were more stable in the RE group. Variability in measured values increased with storage time but decreased with higher EDTA-2K concentration (3 mg/mL). Anticoagulation using CPDA-1 supplemented with EDTA-2K may be appropriate for future EQA programs using fresh canine blood.

## Simple Summary

External quality assessment (EQA) of laboratory analyzers is essential to facilitate comparison of results between institutions. The process requires laboratories using a variety of instruments to analyze the same samples, so that results can be compared and discrepancies between platforms identified. The use of standardized test samples is essential to ensure that the results from each institution are comparable, and this requires guaranteed stability over a specified time period. This study aimed to investigate the use of fresh canine blood samples for EQA of hematology analyzers, specifically evaluating the effects of anticoagulant concentration, storage temperature, and storage duration. Fresh blood samples from five healthy Beagle dogs were collected into CPDA-1 anticoagulant supplemented with EDTA-2K at final concentrations of 1, 2, and 3 mg/mL. Samples were refrigerated or stored at room temperature, and complete blood count measurements were performed using the Microsemi LC-662 point of care hematology analyzer at 2, 5, 8, 24, 48, and 72 h after blood collection. Red blood cell parameters were more stable following refrigeration, while white blood cell and platelet parameters showed greater stability at room temperature. Result variability increased with storage time but decreased with higher EDTA-2K concentration. Overall, refrigerated samples containing CPDA-1 supplemented with EDTA-2K at 3 mg/mL achieved minimal variation in measured values beyond 24 h suggesting that, under appropriate conditions, the use of fresh canine blood may be appropriate for future EQA programs.

## 1. Introduction

Complete blood counts (CBCs) are widely used as routine tests in health screening and clinical diagnosis. Consequently, opportunities to compare test results across multiple facilities are common. In particular, when referring patients from primary to secondary care facilities, it is important to minimize unnecessary repeat testing and provide efficient medical services. To achieve this, it is essential to reduce interlaboratory variability in clinical testing. External quality assessment (EQA) plays a key role in ensuring consistency in test results across facilities [1]. EQA enables laboratories to analyze the same samples using different instruments, compare results, and identify discrepancies among facilities and platforms [2].

In human medicine, automated hematology analyzers are routinely used for blood count screening. In the United States, the International Council for Standardization in Hematology (ICSH); in the United Kingdom, the UK NEQAS for General Hematology; and in Europe, the Hematology Working Group of the European Organization for External Quality Assurance Providers in Laboratory Medicine (EQALM) operate EQA programs in which participating institutions engage in quality assurance activities [1,3,4]. In Japan, several organizations, including the Japan Medical Association and the Japan Association of Clinical Laboratory Technologists, conduct EQA programs [5,6].

A major challenge in blood test EQA is the quality of distributed control materials. For example, in annual EQA programs conducted by the Japan Medical Association and the Japan Society of Clinical Laboratory Technologists, processed blood quality control materials (QCMs) are used. However, cost limitations and matrix effects causing interinstrument variability remain concerns. Few EQA programs use fresh human blood. Although fresh blood does not exhibit matrix effects, it is difficult to prepare in large quantities, and its stability over time is limited [7,8].

In veterinary medicine, hematological QCMs are available for bovine, porcine, equine, and other species; however, no such materials exist for companion animals [9]. Therefore, fresh blood must be used for hematological assessments in companion animal practice. However, EQA using fresh blood is not commonly performed in veterinary medicine. Thus, it is necessary to establish optimal conditions for EQA reference samples using fresh blood. In particular, because blood properties vary between species, it remains unclear whether preparation methods used for human samples apply to canine blood [10,11].

It is preferable for EQA to involve a large number of facilities and to be conducted on a wide scale to standardize test results [1,3,8]. In companion animal veterinary medicine, many facilities perform blood tests as part of routine care. Dogs, in particular, are the most frequently tested species, and they are often referred from primary care facilities to secondary care facilities. Therefore, it is desirable to evaluate and understand interfacility and inter-instrument variations across all facilities, including primary care settings. To this end, it is important to conduct blood EQAs—which involve the regular verification of CBC data obtained from automated blood cell counters at each facility—not only in large secondary care facilities but also in primary care facilities, as part of an effort to standardize laboratory test results.

In primary care veterinary facilities, testing is primarily conducted using point-of-care testing (POCT) devices. The Microsemi LC-662 (LC) automated blood cell counter (HORIBA, Ltd., Kyoto, Kyoto, Japan) for veterinary use is a POCT device that employs the impedance method as its measurement principle and is widely used in general veterinary practice [12]. In our previous study, we evaluated the correlation between the Sysmex XT-2000iV (XT) (Sysmex Corporation, Kobe, Hyogo, Japan), a high-performance automated blood cell counter widely used in large veterinary facilities, and the LC. We demonstrated that the LC is a highly reliable instrument, with LC values showing strong correlation with XT values for all measured parameters in dogs (WBC, RBC, Hb, Ht, MCV, and PLT) [12].

The primary aim of this study was to investigate optimal conditions for conducting EQA using canine blood samples. Fresh canine blood was prepared using CPDA-1 and EDTA-2K as anticoagulants under two storage conditions (room temperature and refrigeration). The Microsemi LC-662 (Horiba, Ltd., Kyoto, Japan), a point-of-care automated hematology analyzer commonly used in clinical settings, was used to evaluate changes in blood count parameters over time. To the best of our knowledge, this combination of anticoagulant conditions has not been investigated for the canine fresh blood to store more than 24 h.

Overall, we evaluated the use of fresh canine blood samples for conducting external quality assessment of hematology analyzers. The effects of anticoagulant type and concentration, storage temperature, and storage duration on complete blood count results were assessed.

## 2. Materials and Methods

### 2.1. Sample Preparation and Measurement Methods

A total of 200 mL of blood was collected from the jugular vein of five healthy Beagle dogs (two males and three females; 5–9 years old) into blood bags containing citrate-phosphate-dextrose-adenine (CPDA-1) anticoagulant (Terumo, Shibuya, Tokyo, Japan). CBC measurements were performed using an in-house hematology analyzer (Microsemi LC-662, Horiba, Kyoto, Japan) according to the manufacturer’s instructions. Each sample was measured in duplicate.

### 2.2. Experimental Conditions

#### 2.2.1. EDTA Supplementation Conditions

Fresh canine blood was transferred from transfusion bags into 50 mL centrifuge tubes containing dipotassium ethylenediaminetetraacetic acid (EDTA-2K) (Fujifilm Wako, Osaka, Japan) at final concentrations of 1, 2, and 3 mg/mL of blood to prepare CPDA-1 blood supplemented with EDTA-2K. The EDTA-2K concentration was set according to Fukuda’s paper [7].

#### 2.2.2. Storage Temperature Conditions

Blood samples were aliquoted into 2 mL cryovials and stored at room temperature (RT group) or refrigerated temperature (RE group) to evaluate the effects of storage temperature. Room temperature was kept between 20 and 24 °C. For the RE group, the refrigerator compartment of a commercial refrigerator-freezer (651S4; Daiwa Reiki Co., Ltd., Tokyo, Japan) was used in this experiment. The temperature was constantly monitored by the built-in thermometer and maintained between 4 °C and 6 °C.

#### 2.2.3. Inter-Individual Variation over Time

To evaluate changes in blood cell counts over time and inter-individual variability, CBC measurements were performed at 2, 5, 8, 24, 48, and 72 h after collection. The first measurement after blood collection was taken 2 h later. This was because it required a series of sample preparation steps, such as aliquoting blood collected from five dogs into multiple samples according to different EDTA-2K concentrations and storage conditions. Samples were inverted 10 times immediately before each measurement. To represent the rate of change over time, with the value measured 2 h after blood collection set as 100%, the graph shows 2 h after blood collection as 0%.

For statistical analysis, we applied a linear mixed-effects model to each hematological test parameter and the EDTA-2K concentration. Regarding storage conditions and storage duration, the interaction between the two was treated as a fixed effect, the dog was treated as a random intercept, and storage duration was treated as a categorical variable. Since this study involved repeated measurements over time using a small sample size, fixed effects were assessed using a Type III *F*-test based on the denominator degrees of freedom calculated using the Kenward–Roger method. Within each storage condition, using the 2 h value as a reference, we applied the multivariate-t method (Dunnett’s test) for multiple comparisons to compare the values at 5, 8, 24, 48, and 72 h. The significance level was set at *p* < 0.05. Statistical analyses were performed using STAT FLEX (version 7; Artech Co., Ltd., Osaka, Japan), while the Brown–Forsythe test was conducted using R (version 4.5.2). Graphs were generated using GraphPad Prism (version 10.6.0; GraphPad Software, San Diego, CA, USA).

#### 2.2.4. Evaluation of Acceptable Limits for Within-Individual Changes over Time Based on the ASVCP Allowable Total Error Criteria for Hematology

Based on the POCT criteria in the ASVCP guidelines Allowable Total Error in Hematology [9], we compared the results of linear mixed-effects model analyses for each measurement parameter with TEa and evaluated the acceptable limits. Given that the analyzer used in this study was an in-hospital POCT hematology analyzer, the acceptable limits were set at 10% for RBC, Hb, and Ht, and 7% for MCV. For WBC and PLT, the limits were set at 20% and 25%, respectively. For each retention time, a point in time was deemed an acceptable time if the rate of change for all five animals fell within the ASVCP tolerance limits for each parameter.

## 3. Results

### 3.1. Rate of Change in CBC over Time According to Storage Temperature

Regarding the effects of time, white blood cell (WBC) values showed an upward trend over time in both the RT and RE groups, with a more pronounced increase in the RE group. In the RT group, PLT levels fluctuated by approximately 10%, showing both increases and decreases; however, in the RE group, PLT levels showed a decreasing trend accompanied by greater fluctuations.

**Figure 1:**
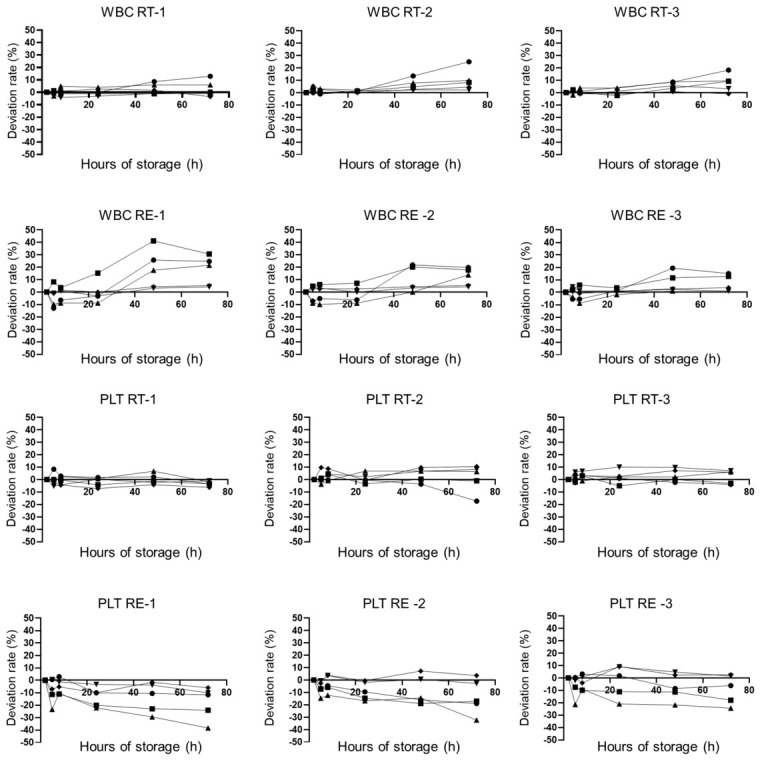

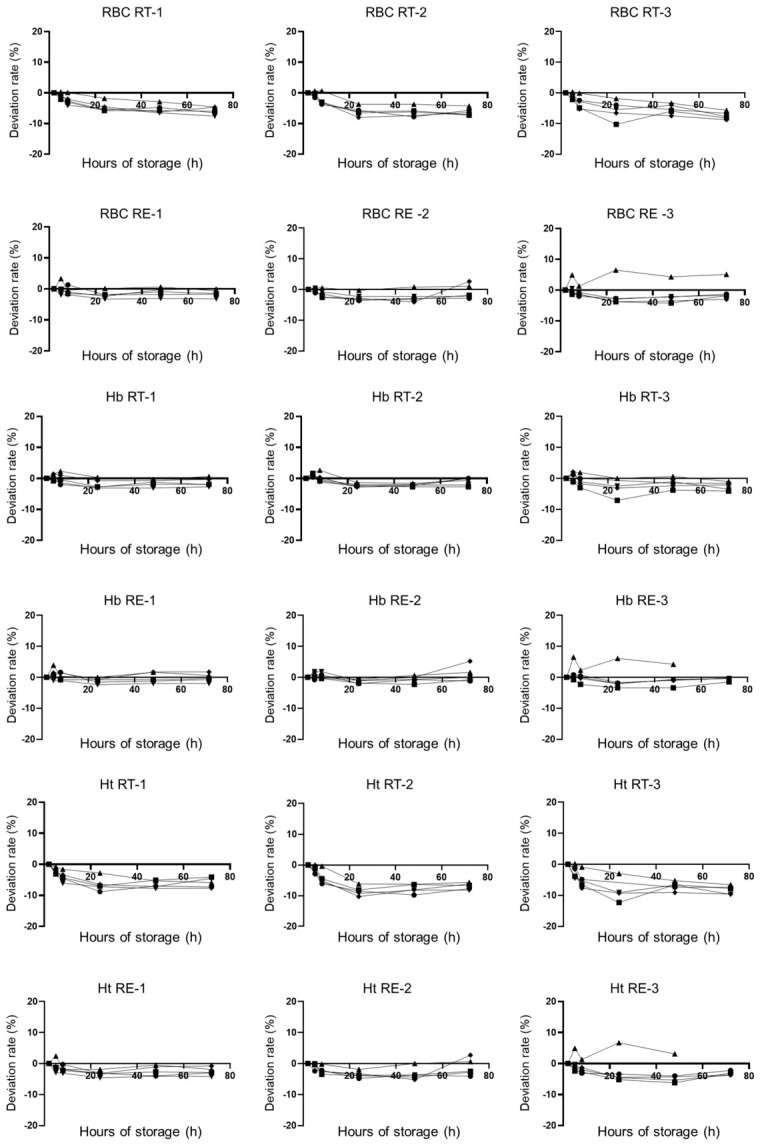

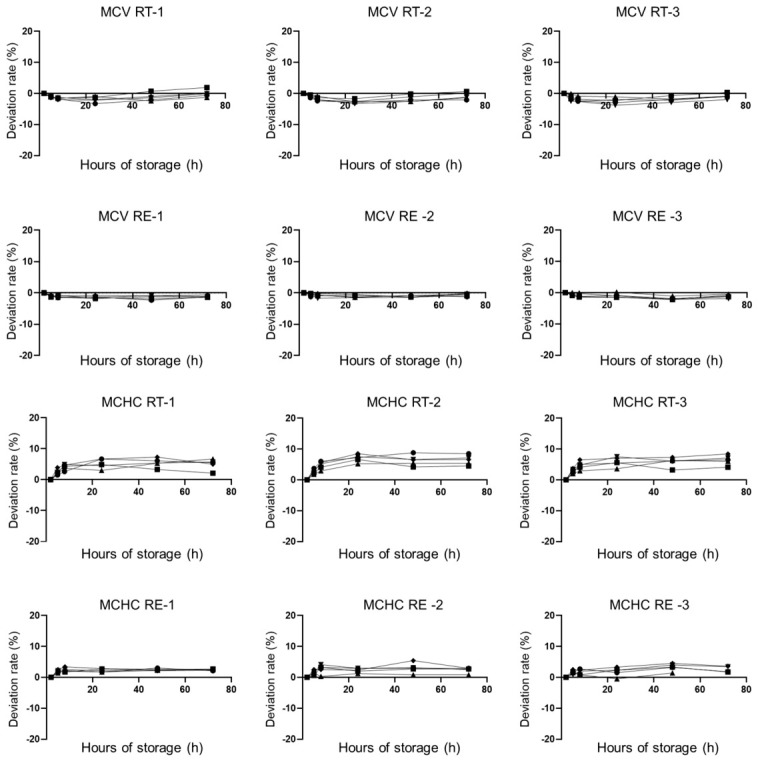
Rate of change in CBC over time: Deviation rates (%) at each measurement time were calculated relative to the value measured 2 h after blood collection (set as 100%), illustrating changes over time (2–72 h after collection) and the effect of temperature. RT-1, 2, 3: room temperature with EDTA concentrations of 1, 2, and 3 mg/mL of blood. RE-1, 2, 3: refrigerated temperature with EDTA concentrations of 1, 2, and 3 mg/mL of blood.

For red blood cell parameters, a decreasing trend was observed over time in the RT group, whereas fluctuations were smaller in the RE group. Hb levels decreased in both the RT and RE groups until 24 h, after which they tended to increase. Similar to RBCs, mean corpuscular volume (MCV) showed a decreasing trend in the RT group. Differences in MCV trends were also observed depending on storage temperature: the RT group showed a decreasing trend up to 24 h followed by an increase from 48 h onward, whereas the RE group showed a pattern in which values decreased up to 48 h and then increased thereafter (Figure 1).

### 3.2. Rate of Change over Time Among Blood Donor Dogs According to Storage Temperature

The storage condition × time interaction was significant for RBC and MCHC at all EDTA-2K concentrations. For parameters other than Hb and PLT, the patterns of change over time differed between room-temperature and refrigerated storage at certain EDTA concentrations. Furthermore, when stored at refrigerated temperatures, no significant changes were observed in Hb at any EDTA concentration for up to 72 h, while for RBC and Ht, no significant changes were observed at an EDTA concentration of 3 mg/dL. MCV showed significant changes when compared to the values after 2 h of storage at room temperature and after 2 h of refrigerated storage (Table 1).

### 3.3. Effects of Temperature Conditions, Storage Time, and Interactions on Hematological Test Parameters

To understand the overall trend in each hematological test parameter, we adjusted the EDTA-2K concentration and performed a linear mixed-effects model analysis with respect to storage time. WBC increased with storage time, and this increase was more pronounced during refrigerated storage. RBC, Hb, and Ht decreased over time, but the rate of decline was reduced during refrigerated storage. While no significant changes over time were detected when PLT was stored at room temperature, a shorter storage period was observed at concentrations of 1 and 2 mg/mL when stored under refrigerated conditions. Regarding MCV, although the “Storage time” was listed as “NS” because it exhibited a nonlinear pattern of change over time, the degree of change was small under refrigerated storage conditions (Table 2).

### 3.4. Acceptable Limits for “Inter-Individual Variability over Time” Using “Total Allowable Error (TEa)”

Based on the ASVCP’s total allowable error criteria, for EDTA-2K at 1 mg/mL stored at room temperature and 3 mg/mL stored refrigerated, all parameters—WBC, RBC, Hb, Ht, MCV, MCHC, and PLT—remained within the acceptable range for up to 72 h. On the other hand, when stored under refrigeration, the storage limit for WBCs and PLTs was short, at 24 h. At a concentration of 2 mg/mL, the storage limits were 8 h for Ht when stored at room temperature and 24 h for WBC when stored refrigerated. When stored at room temperature at 3 mg/mL, the storage limit for red blood cell-related parameters (RBC and Ht) was 8 h. Therefore, a concentration of 1 mg/mL of EDTA-2K for storage at room temperature and 3 mg/mL for refrigerated storage were considered the most stable conditions (Table 3).

## 4. Discussion

In this study, we investigated optimal storage conditions for canine fresh blood using novel anticoagulant combination using CPDA-1 and EDTA-2K. The results suggest that CPDA-1 supplemented with EDTA-2K would be an appropriate anticoagulant for future EQA programs using fresh canine blood, although further study is required to put this into practical use.

In medical practice, the criteria for distribution samples used in blood test EQA include biological equivalence to fresh human blood, safety assurance, minimal changes during storage and transport, stability after opening, sufficient homogeneous volume, ease of preparation, and minimal additives to avoid matrix effects across instruments and reagents [13]. The ideal EQA system aims to provide impartial evaluation of test results by a third-party organization, with participation from as many laboratories as possible across a wide geographical area, thereby reducing interlaboratory variation [14].

When conducting large-scale EQA, processed blood is commonly used due to limitations in blood storage and supply. However, although processed blood exhibits excellent long-term stability, its matrix effects vary depending on the automated blood cell counter [1,7]. For this reason, fresh blood is considered ideal for blood EQA. Although rare, fresh blood surveys have been conducted in human medicine [1,7,8]. However, while fresh blood avoids matrix effects, it presents challenges in terms of long-term sample stability [7].

In companion animal veterinary medicine, processed blood is not available, and blood EQA remains undeveloped worldwide. EQA using fresh blood is still in its early stages, and there is an urgent need to establish standards for anticoagulants in distributed blood samples, acceptable measurement timeframes, and storage conditions.

Although there is no consensus regarding the preparation of fresh human blood samples for EQA, transfused blood is often used due to its longer shelf life. For example, the Osaka Prefectural Medical Association’s Clinical Laboratory Quality Control Survey uses fresh anticoagulated blood as a reference sample for EQA [15]. Samples prepared using EDTA-2K alone tend to show a decline in WBC counts over time, and if not measured within 2–3 days of preparation, WBC values may decrease [16].

CPDA (citrate-phosphate-dextrose-adenine) is a blood preservation solution used for storing red blood cell concentrates. It contains sodium citrate (anticoagulant), sodium dihydrogen phosphate (buffer), glucose (energy source), and adenine (ATP precursor). It is widely used as a base solution for transfusion blood products, enabling long-term storage while maintaining red blood cell function and viability. In veterinary medicine, CPDA-1 is commonly used in blood collection bags for transfusion purposes [17,18,19,20]. CPDA-1 helps maintain higher levels of 2,3-diphosphoglycerate (2,3-DPG) and adenosine triphosphate (ATP), and blood preserved in CPDA can be stored for approximately 35 days for transfusion purposes [21]. Thus, CPDA-1 is an effective anticoagulant for long-term storage of canine blood.

When CPDA-1 is used for canine blood storage, red blood cell counts remain stable for approximately one week, and WBC counts remain stable for approximately four weeks [22]. Compared with CPD, CPDA-1 provides superior preservation, extending red blood cell storage time and improving transfusion quality in veterinary medicine [23]. However, platelet stability differs; studies comparing CPDA-1 and EDTA have shown that citrate, contained in CPDA-1, results in less stable platelet counts than EDTA [24].

In human EQA studies, adding EDTA to CPD transfusion blood reduced platelet decline over time and improved stability of MCV compared with CPD alone or EDTA alone [25,26]. The coefficient of variation (C.V.) for WBC and platelet counts decreased after EDTA was added to CPD, with WBC C.V. falling below 5% and platelet C.V. below 7% [25,26]. Another study comparing CPD with adenine and EDTA reported stable blood count values for up to 5 days [7].

While several reports have examined the effects of time and temperature on canine CBC measurements, most of them use EDTA-anticoagulated blood. When using fresh blood from dogs, it is recommended that samples not be stored for more than 24 h for quality assessment purposes [9,27]. A study conducted in Brazil using canine blood examined the stability of platelet counts using a POCT automated hematology analyzer based on impedance technology, with EDTA-2K as the anticoagulant, at RT and 4 °C. While measurement within 4 h is recommended at RT, samples stored at 4 °C showed no significant changes in measured values up to 6 h after blood collection compared with immediately after collection [28].

In our previous study, which examined CBC measurements using EDTA-2K as an anticoagulant, measured values were more stable at RT than at 4 °C, contrary to the findings reported by Jaguezeski et al. [28]. In that study, we evaluated changes in CBC over time in blood stored at RT and 4 °C using EDTA-2K as an anticoagulant. The results showed that PLT levels in dogs changed significantly over time, beginning to decrease at 12 h under both storage conditions, with a 34% decrease after 24 h at RT and a 57% decrease at 4 °C [12]. The effect of storage appears to differ among hematological parameters depending on storage temperature and analyzer type [29].

Furthermore, in the present study, which used an anticoagulant consisting of CPDA-1 and EDTA-2K, both platelet and WBC counts showed more stable temporal changes at RT than under refrigerated (RE) conditions (Figure 1). In contrast, red blood cell counts were more stable under RE conditions (Figure 1). Since RT varies by region and season, its effect on measurement values also differs. Refrigerated storage has the advantage of suppressing cellular activity; therefore, it may be more suitable for distributing samples over long distances. Accordingly, refrigerated storage may be preferable for EQA of blood cells in veterinary medicine in the future.

Regarding temporal changes in CBC, previous reports on canine blood have examined these changes using either EDTA or CPDA-1. This study represents the first attempt worldwide to investigate the use of EDTA-2K added to CPDA-1. Compared with previous studies using EDTA alone, similar trends were observed, with platelet (PLT) counts decreasing and WBC counts increasing over time. However, in this study, the C.V. for both WBC and PLT was lower when CPDA was combined with EDTA. These results suggest that CPDA with added EDTA is less susceptible to time-dependent effects than EDTA alone and is therefore useful for large-scale blood surveys.

Unlike previous studies, MCV showed a decreasing trend up to 24 h, followed by an increasing trend [12]. This suggests that red blood cell swelling induced by EDTA may be suppressed by CPDA components. These findings are consistent with those of Fukuda et al., who reported similar stability patterns in fresh human blood using a comparable anticoagulant system (Figure 1) [7]. Because variability in test results increases over time after blood collection, inter-assay variability should be interpreted in relation to time elapsed since sampling when conducting EQA (Table 1 and Table 2).

With regard to EDTA, it has been reported in humans that it affects neutrophil morphology over time, with stronger effects observed at higher concentrations. Studies using EDTA-2K with automated analyzers (XE-5000, ADVIA 2120i, and DxH 800) have shown decreased WBC counts and increased MCV over time [7]. Fukuda et al. [7] investigated optimal EDTA concentrations added to CPDA and reported no effect on WBC, PLT, or MCV at concentrations of 0.5 to 2.8 mg/mL blood.

In the present study, EDTA-2K was evaluated at concentration equivalent to routine EDTA (1–2 mg/mL [30]) as well as at higher concentration at 3 mg/mL. Higher EDTA concentrations tended to reduce variability in WBC and RBC measurements (Table 1). Therefore, when conducting large-scale EQA and using refrigerated storage to minimize regional temperature variation, higher EDTA concentrations may help reduce fluctuations in WBC counts. In contrast, platelet variability was not affected by EDTA concentration (Table 1).

In contrast to Kondo et al., who reported that adding EDTA to CPD solution in human blood suppressed declines in WBC and platelet counts and maintained C.V. values within 5% and 7%, respectively [25,26]., the present study observed platelet fluctuations of up to 20% under cooling conditions. This suggests that canine blood may be more prone to platelet agglutination, highlighting the need for species-specific guidelines for canine blood use. It has been reported that dog blood tends to clot more easily than human blood [11]. In this study it was found that adding EDTA-2K to CPDA-1 resulted in a lower rate of variation over time compared to when the sample was preserved with EDTA-2K alone [12]. Based on these findings, the use of an anticoagulant containing CPDA-1 and EDTA-2K for blood preservation was considered a useful method for EQA using fresh canine blood.

ASVCP provides total allowable error (TE_a_) recommendations for commonly analyzed hematology measurands for veterinary personnel [9]. Based on the ASVCP’s TEa criteria, blood storage at room temperature in EDTA-2K at 1 mg/mL and refrigerated storage at 3 mg/mL were within the acceptable range for up to 72 h (Table 3). For the future EQA using canine fresh blood, refrigerated storage may be preferable in that it can mitigate the effects of temperature variations across seasons and regions. If refrigerated storage is chosen, the condition of adding 3 mg/mL of EDTA-2K to CPDA-1 is considered to provide the greatest stability.

Limitations of this study include the small sample size, a restricted number of canine species, limited variation in anticoagulants, and a narrow range of EDTA concentrations. First, the sample size was small, making it difficult to sufficiently assume the normality of the data. Secondly, this study focused on Beagles (5–9 years old), which are the most widely used breed in various studies; however, the potential influence of dog breed, age, or individual biological variation on the observed patterns of stability cannot be ruled out. Finally, anticoagulant types are limited, and EDTA concentrations could only be tested under few variations which are similar to those reported in previous studies, so further investigation is necessary to establish optimal EQA sample conditions. Therefore, it is considered necessary to conduct a similar analysis in the future using increased numbers and more diverse canine populations, ages, as well as wide variety of anticoagulant conditions to achieve EQA in the field of veterinary medicine.

## 5. Conclusions

For EQA testing of canine blood counts, appropriate conditions for blood samples stored for more than 24 h are needed. We propose that anticoagulant using CPDA-1 supplemented with EDTA-2K would be appropriate for future EQA programs using fresh canine blood but this requires further validation. Blood cell parameters of canine blood anticoagulated with CPDA-EDTA-2K were found to be temperature-dependent; while WBC and PLT counts tended to remain stable at RT, RBC parameters tended to remain stable in the refrigerated condition. For further study, it is necessary to examine the temperature and EDTA concentration conditions for EQA in greater detail.

## Figures and Tables

**Table 1:** Interaction between storage temperature conditions and storage time at various EDTA concentrations, and comparison with the 2 h values for each storage condition: The comparisons with the 2 h reference values were derived from the results of Dunnett-type multiple comparisons.

		Temperature Condition × Time Interaction		Storage (RT) Comparison with 2-h Values	Storage (RF) Comparison with 2-h Values
Parameter	EDTA-2K Concentration (mg/mL)	*F* Value (*df*:5, 44)	*p* Value	First Significant Time Point	First Significant Time Point
WBC	1	4.1	<0.01	ND	48
	2	0.6	0.69	72	72
	3	0.3	0.91	72	ND
RBC	1	9.9	<0.001	8	24
	2	10.6	<0.001	8	24
	3	5.3	<0.01	8	ND
Hb	1	0.4	0.87	ND	ND
	2	2.0	0.10	24	ND
	3	1.7	0.158	24	ND
Ht	1	5.0	0.001	5	8
	2	9.7	<0.001	8	24
	3	4.6	0.002	8	ND
MCV	1	3.0	0.022	8	5
	2	3.2	0.015	8	8
	3	5.5	<0.001	5	5
MCHC	1	5.4	<0.001	5	5
	2	11.9	<0.001	5	5
	3	10.5	<0.001	5	5
PLT	1	2.4	0.053	ND	24
	2	1.9	0.109	ND	48
	3	1.4	0.232	ND	ND

ND indicates that no statistically significant difference from the 2 h reference was detected through 72 h after multiplicity adjustment. df: degree of freedom.

**Table 2:** The Effects of Temperature Conditions, Storage Time, and Interactions on Hematological Parameters. The “Temperature condition” section shows significant differences between storage temperatures, while the “Storage time” section shows changes in measured values over time. “Direction of change” indicates the trend of increase or decrease. An interaction between temperature and storage time indicates that the rate of change over time differs between room temperature and refrigerated storage conditions.

Parameter	Temperature Condition	Storage Time	Direction of Change	Temperature Condition × Storage Time	Interpretation
WBC	*p* < 0.001	*p* = 0.005	↑	*p* = 0.021	WBC levels increased over time, and this increase was more pronounced during refrigerated storage.
PLT	*p* < 0.001	NS		*p* = 0.001	PLT exhibited changes over time that were dependent on refrigerated storage.
RBC	*p* < 0.001	*p* < 0.001	↓	*p* < 0.001	The RBC count decreased over time, but this decline was mitigated during refrigerated storage.
Hb	NS	*p* < 0.001	↓	*p* = 0.007	Hb levels decreased over time, but this decline was mitigated by refrigerated storage.
Ht	*p* < 0.001	*p* < 0.001	↓	*p* < 0.001	Ht varied depending on temperature conditions and decreased over time, but this decrease was mitigated under refrigerated storage conditions.
MCV	*p* < 0.001	NS		*p* = 0.014	MCV showed changes over time that were dependent on refrigerated storage
MCHC	*p* < 0.001	*p* < 0.001	↓	*p* < 0.001	MCHC increased over time, but this increase was mitigated under refrigerated storage conditions.

↑ and ↓ indicate statistically significant increases and decreases, respectively, from the baseline value; NS indicates no statistically significant difference from baseline.

**Table 3:** Maximum Acceptable Storage Times for Canine Hematologic Measurands Based on ASVCP Primary Total Allowable Error Criteria at Different EDTA-2K Concentrations and Storage Temperatures: Each condition cell shows the maximum allowable storage time in hours.

		1 mg/mL		2 mg/mL		3 mg/mL	
Analyte	Primary TEa (%)	Room Temperature	Refrigerated	Room Temperature	Refrigerated	Room Temperature	Refrigerated
WBC	20	72	24	48	24	72	72
RBC	10	72	72	72	72	8	72
HGB	10	72	72	72	72	72	72
HCT	10	72	72	8	72	8	72
MCV	7	72	72	72	72	72	72
MCHC	10	72	72	72	72	72	72
PLT	25	72	24	72	48	72	72

## Data Availability

The datasets generated and/or analyzed during the current study, including additional hematological data and statistical analyses, are available from the corresponding author upon reasonable request.

## Abbreviations

The following abbreviations are used in this manuscript:

C.V.	Coefficient of variation
CBC	Complete blood counts
CPDA	Citrate-phosphate-dextrose-adenine
DOI	Digital object identifier
EQA	External quality assessment
EQALM	External Quality Assurance Providers in Laboratory Medicine
MCV	Mean corpuscular volume
ICSH	International Council for Standardization in Hematology
POCT	Point-of-care testing
QCM	Quality control materials
RT	Room temperature
